# Brazilian experience with atopy patch tests for *Dermatophagoides pteronyssinus*, *Dermatophagoides farinae* and *Blomia tropicalis*

**DOI:** 10.1186/s40413-018-0206-3

**Published:** 2018-10-24

**Authors:** Ingrid Pimentel Cunha Magalhães de Souza Lima, Beatriz Julião Aarestrup, Eduardo Magalhães de Souza Lima, Marina Cunha de Souza Lima, Eduardo Cunha de Souza Lima, Fernando Monteiro Aarestrup

**Affiliations:** 1 Federal University of Juiz de Fora, Juiz de Fora, Brazil; 2 Faculty of Medical Science and Health SUPREMA, Juiz de Fora, Brazil; 3 Centro Universitário de Belo Horizonte – UniBH, Belo Horizonte, Brazil; 4 Pontifícia Universidade Católica de Minas Gerais - Campus de Betim, Betim, Brazil

**Keywords:** Atopy patch test, Skin prick test, House dust mite, *Dermatophagoides pteronyssinus*, *Dermatophagoides farinae*, *Blomia tropicalis*, Rhinitis, Asthma, Atopic dermatitis

## Abstract

**Background:**

The aim of this study was to evaluate the positivity rates of atopy patch tests for *Dermatophagoides pteronyssinus*, *Dermatophagoides farinae* and *Blomia tropicalis* in patients with respiratory diseases such as asthma and allergic rhinitis with or without atopic dermatitis.

**Methods:**

The patients’ clinical histories were collected, and the patients were subjected to skin prick and patch tests with the three different house dust mites on the same day. The patch tests were examined 48 hours later, and then patients were divided into two groups: I- patients with respiratory diseases, such as asthma and/or rhinitis, and atopic dermatitis and II-patients with only respiratory diseases. A total of 74 patients ranging in age from 2 to 60 years were included in this study; 16 patients were included in group I and 58 were included in group II. This study was approved by the human ethics committee of the Faculty of Medical Science and Health SUPREMA (number 2.007.135), and written informed consent was collected from each patient or their parents prior to enrollment.

**Results:**

In the skin prick tests, the most prevalent mite that evoked a reaction was *Dermatophagoides pteronyssinus*, followed by *Dermatophagoides farinae* and *Blomia tropicalis*. Regarding the atopy patch tests, the mite that most frequently induced a positive reaction was *Dermatophagoides farinae* (78.4%), followed by *Dermatophagoides pteronyssinus* (77%) and *Blomia tropicalis* (52.7%). A comparison of the skin prick and atopy patch tests revealed that 53 patients (71.6%) were positive on both tests, and 30 (56.6%) patients were positivite for the same mite. We found six patients (8%) who had a positive clinical history of allergy and only exhibited positivity on the atopy patch test.

**Discussion:**

Most studies have been performed with atopic dermatitis patients, but in this study, most of the patients had respiratory conditions. *Blomia tropicalis *is a mite that is prevalent in tropical areas, such as Brazil, and only two publications include these three mites, wich are present in Brazil. The APT may produce positive results in concordance with the SPT resuts, but may also be the only positive test ( 8%) as we observed in our study. These results suggest that the mite atopy patch test is relevant and should be considered as an additional test for patients with clinical histories of allergic respiratory disease who have negative prick test results.

**Conclusion:**

The APT should be considered as an additional test when the SPT and specific serum IgE tests are negative in patients with clinical histories of allergies.

## Background

The first use of aeroallergen extracts for skin patch testing was reported in 1937 [1]. In 1982, Mitchell et al., performed the first patch test with purified mite antigen in atopic dermatitis patients and confirmed that acute eczematous lesions can be induced by the application of inhalant allergens to the skin. This technique was defined as the atopy patch test (APT), introduced in 1989 by Ring et al., [2], because this test can be applied to subjects with atopic eczema. Many studies [3–7] have been performed to standardize the APT in terms of single allergen concentrations, optimal vehicles, and the time intervals required to evaluate the results, with the aim of providing a reproducible tool that can be used in routine diagnostic allergy workups. According to the European Task Force on Atopic Dermatitis, a standardized APT technique requires purified allergen preparations measured in biological units or in major allergen content, the use of 12-mm Finn-Chambers on Scanpor tape, the use of petrolatum as the vehicle, and reading of the results at 48 and 72 hours [8]. The APT has been determined to be relevant for foods, especially in children, but standardization is lacking and the reproducibility of this test is poor [9].

Regarding allergy testing, the skin prick test (SPT) and measurements of specific immunoglobulin E (IgE) antibodies in the serum are used to indicate sensitization in immediate type I hypersensitivity reactions, but these tests must be combined with the subject’s case history to diagnose a clinical allergy. The APT, which consists of applying the suspected allergen to the skin using the same method as patch testing for contact dermatitis, was introduced as a technique for evaluating sensitization to aeroallergens and measuring type IV sensitization in subjects with atopic dermatitis [2]. This test was later confirmed to be a valid test, particularly for dust mites [4, 5, 16–18]. Notably, recent studies have found that the APT may be the only positive test in patients with atopic dermatitis and respiratory allergies [19, 20].

House dust mites are a major cause of allergic diseases worldwide [10]. The most frequently responsible mites are *Dermatophagoides pteronyssinus* and *Dermatophagoides farinae*, which produce efficient allergens that are able to induce sensitization and clinical disease [11]. The spectrum of allergic manifestations includes atopic dermatitis [12] (which is particularly frequent in children), rhinitis, asthma [13, 14] and, very rarely, anaphylaxis [15]. Mites of the genus *Blomia*, including *Blomia tropicalis*, are an important cause of IgE antibody responses among asthmatic patients in tropical and subtropical areas of the world [21–23]. Exposure to *Blomia tropicalis* has been documented in houses in Brazil, and this allergen has been designated Blo t 5. *Dermatophagoides pteronyssinus, Dermatophagoides farinae* and *Blomia tropicalis* are currently the main causes of allergic diseases in Brazil, and our patients are tested for allergies to these mites with the SPT in our daily practice.

In the past two decades, research has demonstrated that in patients with respiratory diseases such as rhinitis and asthma, allergic symptoms may be sustained by T-cell-mediated reactions as demonstrated by positive results in the APT. Importantly, in patients with T-cell-mediated allergies, the APT can be the only positive test [16, 20, 25, 26]. These clinical data on the role of the APT are supported by evidence regarding the capacity of this test to reproduce the pathophysiological events of atopic dermatitis. Specifically, application of the APT to the skin of atopic dermatitis patients is followed by an influx of inflammatory dendritic epidermal cells [27], which enables the detection of a shift from TH2 cytokines to a TH1 pattern that can be detected 24 hours after the APT, as occurs for chronic atopic dermatitis skin lesions after 48 hours [28, 29].

We performed a study that applied methodologies that have been used in Europe in recent years, especially those of Fuiano et al., [26, 33, 35]. We replicated these previous studies to evaluate the potential use of the APT as an additional test, especially for patients with positive histories of allergic diseases with negative SPT and radioallergosorbent test (RAST) results. We used the three most common house dust mites in Brazil*, Dermatophagoides pteronyssinus, Dermatophagoides farinae and Blomia tropicalis.*

## Methods

A total of 74 patients ranging in age from 2 to 60 years were included in this study and were divided into the following 2 groups: group I - patients with atopic dermatitis and other allergic respiratory diseases, such as allergic rhinitis and/or asthma; and group II - patients with only respiratory diseases, such as rhinitis and/or asthma, without atopic dermatitis.

Atopic dermatitis was diagnosed according the criteria of Hanifin and Rajka [30], and allergic rhinitis and asthma were diagnosed according to the *Allergic Rhinitis and its Impact on Asthma* (ARIA) guidelines [31].

The patients were subjected to SPT with extracts from International Pharmaceutical Immunology ASAAC Brasil (IPI ASAAC) and to APTs with aeroallergens from *Dermatophagoides pteronyssinus, Dermatophagoides farinae, Blomia tropicalis* from IPI ASAAC Brasil at concentrations of 0.085 g ± 10% on the same day. A negative control of saline solution and a positive control with histamine were used for the SPT. The reactions were considered positive in the presence of a wheal diameter of at least 3 mm larger than that observed for the negative controls.

For the APT, the substances were applied to the intact skin of the lower back and held firmly in position using a Finn Chamber (Smart Practice, 3400 E Mc Dowell Rd., Phoenix, Arizona, USA) 12 mm in diameter on a micropore tape. The application period was 48 hours, and the test was examined at least 30 minutes after removal to avoid any margin effect. Only one evaluation was performed after 48 hours, as suggested by Darsow et al., [4] in 1995. The results were interpreted according to the American Academy of Dermatology APT criteria using a scale ranging from 1+ (weak reaction) to 3+ (strong reaction) [32]. Finn Chambers with petrolatum were used in the control test. We informed the families that they should avoid using antihistamines and oral or topical corticosteroids for seven days prior to the SPT and APT.

The study was approved by the human ethics committee of the Faculty of Medical Science and Health SUPREMA (number 2.007.135), and written informed consent was obtained from each patient or their parents prior to enrollment.

Patients who had taken antihistamines, oral or topical corticosteroids or immunosuppressive drugs that might have altered the positivity of the tests, patients with food allergies; those with gastrointestinal symptoms, such as eosinophilic esophagitis; children under 2 years of age; and patients over 60 years were excluded from the study.

## Results

We performed SPTs and APTs on 74 patients using three different house dust mites: *Dermatophagoides pteronyssinus, Dermatophagoides farinae and Blomia tropicalis*. Fifty-three patients were female (71.6%), and 21 were male (28.4%). Group I comprised 16 patients (patients with atopic dermatitis and other allergic respiratory diseases, such as allergic rhinitis and/or asthma), and 58 patients were included in group II (patients with only respiratory diseases, such as rhinitis and/or asthma, without atopic dermatitis) (Table 1).

**Table 1:** SPTs and APTs performed in 74 patients

	Variables	Number	Percentage (%)
Sex	Male	53	71.6%
	Female	21	28.4%
Group	I	16	21.6%
	II	58	78.4%
Total		74	100%

**Fig. 1:**
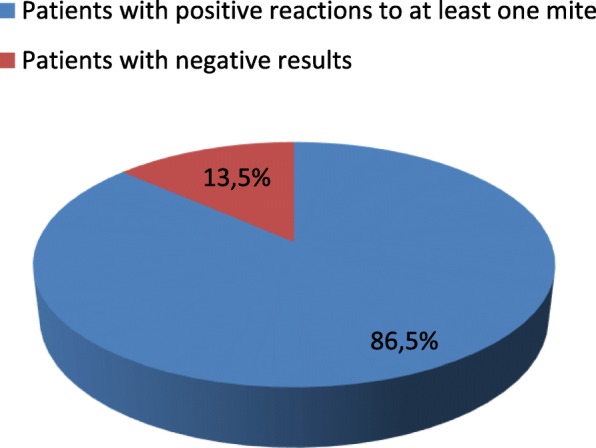
Total SPT positivity

**Fig. 2:**
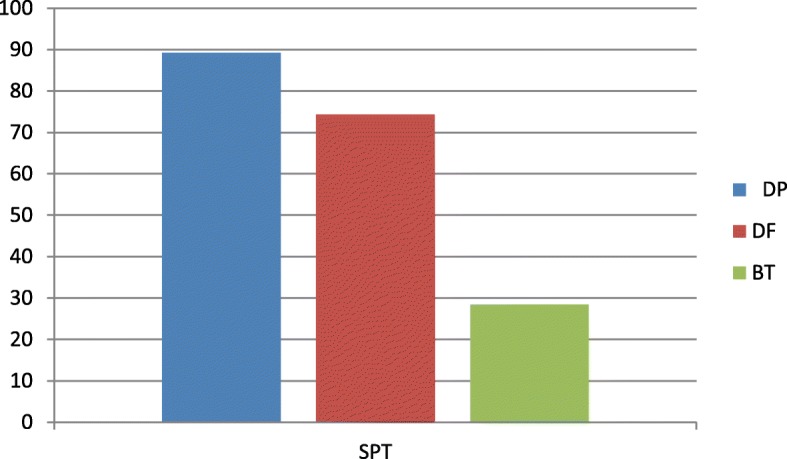
Positive results in the SPTs for each mite including: *Dermatophagoides pteronyssinus (89.2%), Dermatophagoides farinae (74.3%) and Blomia tropicalis (28.4%)*

**Fig. 3:**
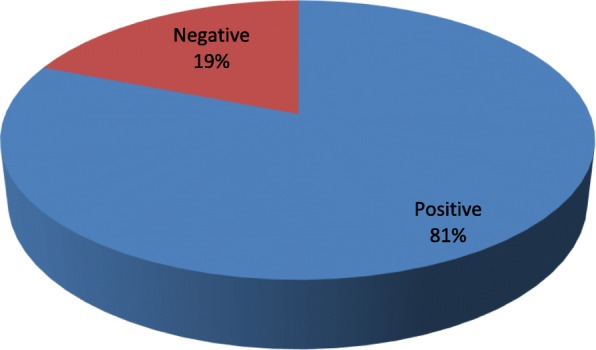
Total APT positivity for all mites tested

**Fig. 4:**
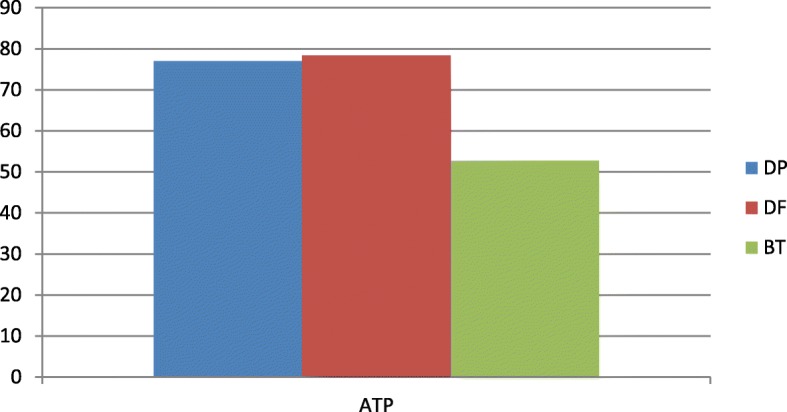
APT positivity for each mite: *Dermatophagoides pteronyssinus* (77%), *Dermatophagoides farinae* (78.4%), *Blomia tropicalis* (52.7%)

In the SPTs, 64 patients were positive for at least one mite (86.5%) (Fig. 1). The most frequently positive mite was *Dermatophagoides pteronyssinus* (66 patients, 89.2%), followed by *Dermatophagoides farinae* (55 patients, 74.3%) and *Blomia tropicalis* (21 patients, 28.4%) (Fig. 2). In the APT, we observed 60 positive patients (81%) and 14 negative patients (19%) (Fig. 3). Most of the positive patients were positive for more than one mite, and only 2 patients were positive for only *Dermatophagoides pteronyssinus.* Fifty-seven patients were positive for *Dermatophagoides pteronyssinus* (77%), 58 were positive for *Dermatophagoides farinae* (78.4%) and 39 were positive for *Blomia tropicalis* (52.7%) (Fig. 4).

**Fig. 5:**
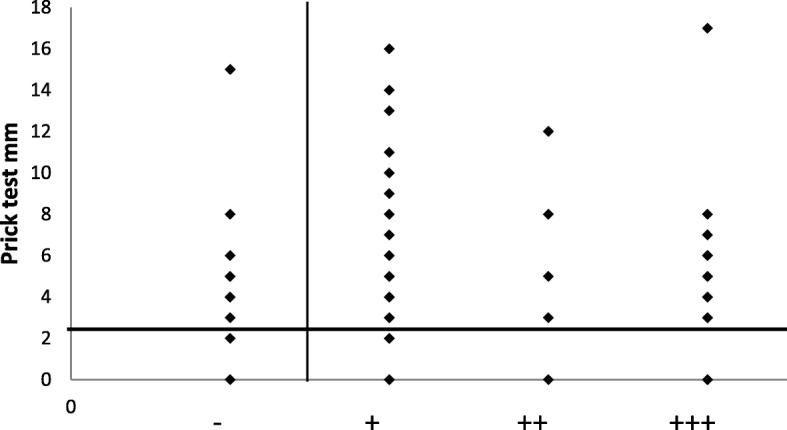
Points located in the upper right quadrant were positive in both tests, i.e., the prick test measurements were greater than 3 mm and the patch tests exhibited some degree of positivity for at least one of the mites

**Fig. 6:**
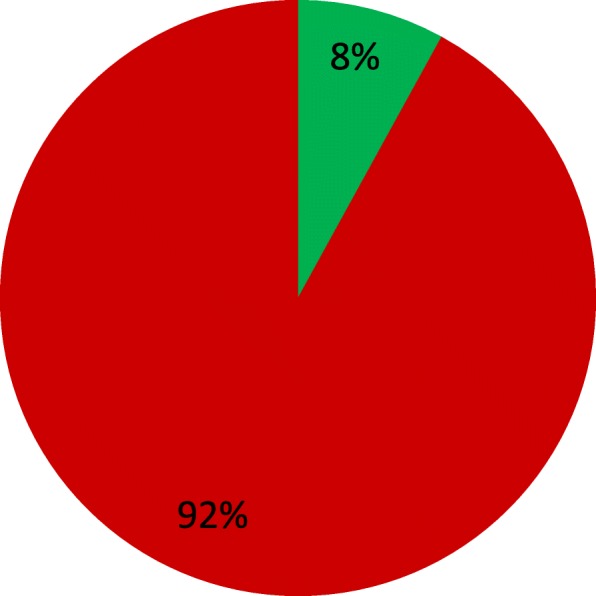
Patients who were positive in the APT and negative in the SPT (6 patients, 8%)

When we compared the positivity between the SPT and APT, 53 patients (71.6%) were positive in both tests. Of those, 30 patients (56.6%) exhibited positivity to the same mite (Fig. 5). We found that 6 patients (8%) had negative SPT and positive APT results (Fig. 6). Of those, *Dermatophagoides pteronyssinus* and *Dermatophagoides farinae* were positive for all 6 patients, including 5 + and one ++ positive finding. *Blomia tropicalis* exhibited positivity in 3 patients ranging from + to +++. No positive results were obtained for negative control tests in the SPT or APT. Moreover, the histamine control yielded all positive results in SPTs.

## Discussion

The SPT is the most frequently performed test for assessing immediate hypersensitivity (type I) for both respiratory and skin allergies. The APT evaluates allergen-induced delayed type hypersensitivity (type IV). Eczematous reactions at the site of application are examined 48–72 hours later to determine the sensitization of patients to the allergen [16].

Positive patch tests are associated with the presence of IgE bearing Langerhans cells in the epidermis of atopic dermatitis patients [36, 37]. Aeroallergens bind to Langerhans cells and activate allergen-specific T-cell responses, which are associated with IgE production and delayed type hypersensitivity. A large body of evidence indicates the role of APT in the evaluation of aeroallergens in atopic dermatitis, but its relationships to the SPT and specific IgE levels in respiratory allergies have not been fully investigated.

Several articles have demonstrated the relevance of aeroallergens in patients with atopic dermatitis as evaluated by the SPT and APT, and avoidance of these aeroallergens results in improvements in dermatitis [38–40]. In one study, the sensitization rate to house dust mites was 56% according to the RAST, 24% for the SPT and 47% for the APT in patients with atopic dermatitis [41].

Guler et al., [16] tested sensitivity to *Dermatophagoides pteronyssinus* with an APT in 63 patients with respiratory diseases, including asthma and rhinitis, and found a 25% positivity rate (30% among asthmatic children and 20% among children with allergic rhinitis). No correlation with the SPT papule diameter was found, and APT reactions were minimal (+). This finding corroborates the finding in our study, which may imply a role of delayed hypersensitivity in patients with respiratory allergies.

In recent decades, epidemiological studies of allergic disorders in children, including atopic dermatitis, rhinitis and asthma, have demonstrated continuous increases in prevalence. However, these studies have typically been performed with questionnaires and, occasionally, with SPTs or in vitro IgE tests, and the portions of these allergies sustained by a cell-mediated mechanism were neglected because an essential test, i.e., the APT, was not performed [33].

Several studies have attempted to standardize the APT for aeroallergens with a wide variety of methods [4, 5, 19, 24]. Skin abrasion [45–47], tape stripping [18, 48], and sodium lauryl sulfate (SLS) application [49] are frequently used to enable allergen penetration. However, studies of APTs on nonabraded, nonpretreated skin have also been performed [2, 50–52] and produced different numbers of positive reactions; obviously, these differences were partially related to different allergen contents in the preparations. Patch tests have been performed with three lyophilized common aeroallergens: house dust mite (*Dermatophagoides pteronyssinus*), cat dander, and grass pollen (Allergopharma, Reinbek, Germany). At the time of removal, after 48 hours, 57 areas developed positive reactions, and 49 areas were graded +, but after 72 hours, the number of positive reactions declined to 41. Compared with the reactions to classic patch tests with contact allergens, APT reactions showed a different time course, and the peak severity of APT reactions was reached after 48 hours. After this time, the reactions showed no further increase but rather decreased, indicating differences between these reactions and responses to classic contact allergy tests. These allergens were used at concentrations of 1000 protein nitrogen units (PNU)/g and 10,000 PNU/g in two different vehicles: (1) white petrolatum/10% isopropyl myristate and (2) methylcellulose hydrogel/10% propylene glycol. No differences were observed with the vehicle used, but the higher concentration elicited more positivity than the lower concentrantion [4]. Various concentrations of allergens in APTs are described in the literature, with a range from 1× SPT (10,000 AU/ml) to 1.000× SPT [4]. Van Voorst Vader et al. concluded that the optimal allergen concentration is 500× SPT with an exposure time of 48 hours [18]. Langeveld-Wildschut et al. concluded that the concentration should be equal to 1× SPT, and according to their results, increasing the allergen concentration to 10× SPT did not significantly influence the number of positive results [42]. Authors from Poland also studied the influence of allergen concentrations and found that 0.1× SPT was too low, while 10× SPT produced significantly more reactions than 1× SPT [43]. Data in the literature indicate that positive APT reactions can occur in 15–90% of atopic eczema patients depending on the methodology used for testing [43, 44].

Liu Y et al. performed a systematic review and meta-analysis last year, comparing APT to SPT for the diagnosis of mite-induced atopic dermatitis. In the ten studies analyzed, the percentage of APT- positive subjects ranged from 14 to 70%, likely due to the lack of standardized techniques, different allergen sources and purification in both the APT and SPT tests. The majority of studies used *Dermatophagoides pteronyssinus,* and few studies used other mite species. Different Finn chamber sizes, extracts and vehicles were used in different studies, and various methods were used to score APT results. The meta-analysis concluded that APT should be used alongside SPT for the identification of mite sensitization in patients with atopic dermatitis, but better standardization would be valuable.

The limitations of APTs include this lack of standardization. Our study was performed with APTs for aeroallergens from *Dermatophagoides pteronyssinus, Dermatophagoides farinae and Blomia tropicalis* from IPI ASAAC at concentrations of 0.085 g ± 10%, as described in the Fuiano et al. study. Positivity to mites in the APT among patients with respiratory symptoms was higher than other values found in other studies, and we hypothesized that *Dermatophagoides pteronyssinus, Dermatophagoides farinae* and *Blomia tropicalis* are the main sources of allergies in Brazil and produce the greatest positivity rates in SPTs, which differs from observations in the USA and Europe. It is difficult to compare these studies because each laboratory and each country have different methods of standardization.

In our study, the percentage of patients with a positive allergy history, a negative SPT and a positive patch test was 8%. Data in the literature suggest that the APT is positive in 17.5% of subjects with a positive history to only inhalant allergens. Most studies of APT positivity have been performed in atopic dermatitis patients; we obtained a different result in our study. No difference in reactivity was observed in the group with atopic dermatitis and respiratory symptoms relative to the group with respiratory symptoms alone.

Among different allergy mechanisms, the T-cell mediated mechanism has long been recognized as decisive in atopic dermatitis; thus, the APT has a significant diagnostic role, as initially demonstrated by sensitization to foods [32], and later demonstrated by sensitization to inhalant allergens, particularly house dust mites [33, 34]. The fact that the APT may be the only positive test in patients with respiratory allergies underlines the importance of the inclusion of this test in the diagnostic work-up of allergy patients. Otherwise, patients with negative results who undergo an SPT or in vitro IgE tests may be erroneously classified as nonallergic or as having local rhinitis. Thus, the APT should be considered as an additional method for diagnostic application.

## Conclusions

The results suggest that the APT is relevant to clinical investigations in patients with respiratory allergic symptoms, especially when they have a clinical history and negative SPT and/or IgE results specific to mites. The APT may produce positive results in concordance with the SPT results but may also be the only positive test (8%), as we observed in our study. The APT should be considered as an additional test when the SPT and specific serum IgE tests are negative in patients with typical clinical histories of allergies.

## Abbreviations

AD: Atopic dermatitis
APT: Atopy patch test
BT: *Blomia tropicalis*
DF: *Dermatophagoides farinae*
DP: *Dermatophagoides pteronyssinus*
HDM: House dust mite
SPT: Skin prick test
